# Effects of hypersplenism on the outcome of hepatectomy in hepatocellular carcinoma with hepatitis B virus related portal hypertension

**DOI:** 10.3389/fsurg.2023.1118693

**Published:** 2023-03-20

**Authors:** Xiao Chen, Dong Wang, Rui Dong, Tao Yang, Bo Huang, Yanlong Cao, Jianguo Lu, Jikai Yin

**Affiliations:** Department of General Surgery, Tangdu Hospital, Fourth Military Medical University, Xi’an, China

**Keywords:** hypersplenism, prognosis, hepatitis B virus, hepatectomy, hepatocellular carcinoma ( HCC)

## Abstract

**Background:**

Although hepatectomy plus splenectomy is not regularly recommended for hepatocellular carcinoma (HCC) with portal hypertension related hypersplenism due to the high risk accompanied with surgical procedures for now. Many researchers still believe that hypersplenism is a controversial adverse prognostic factor for HCC patients. Thus, the primary objective of the study was to determine the effects of hypersplenism on the prognosis of these patients during and after hepatectomy.

**Methods:**

A total of 335 patients with HBV-related HCC who underwent surgical resection as primary intervention were included in this study and categorized into three groups. Group A consisted of 226 patients without hypersplenism, Group B included 77 patients with mild hypersplenism, and Group C contained 32 patients with severe hypersplenism. The influence of hypersplenism on the outcome during the perioperative and long-term follow-up periods was analyzed. The independent factors were identified using the Cox proportional hazards regression model.

**Results:**

The presence of hypersplenism is associated with longer hospital stays, more postoperative blood transfusions, and higher complication rates. The overall survival (OS, *P* = 0.020) and disease-free survival (DFS, *P* = 0.005) were significantly decreased in Group B compared to those in Group A. Additionally, the OS (*P* = 0.014) and DFS (*P* = 0.005) were reduced in Group C compared to those in Group B. Severe hypersplenism was a significant independent prognostic variable for both OS and DFS.

**Conclusion:**

Severe hypersplenism prolonged the hospital stay, increased the rate of postoperative blood transfusion, and elevated the incidence of complications. Furthermore, hypersplenism predicted lower overall and disease-free survivals.

## Introduction

Hepatocellular carcinoma (HCC) is the most found histological type of liver cancer worldwide. The geographical distribution of the incidence of HCC greatly varies in different locations. A high burden of disease is observed in endemic areas where the infection of hepatitis B virus (HBV) is common and in people with chronic liver disease and cirrhosis, especially in China. A majority of liver cancers occur in Asia and China accounts for 47% of all liver cancers globally (1, 2). HBV-related HCC always lead to a poor prognosis, meanwhile portal hypertension (PH) related hypersplenism is a common manifestation of HCC, leading to leucopenia, thrombocytopenia, and anemia. Advanced cases of HCC with end-stage liver diseases are recommended for liver transplantation (3). Nevertheless, due to the high cost and shortage of donors, liver transplantation is controversial for patients with hypersplenism and an adequate hepatic reserve. Hypersplenism can also lead to hepatic damage, increase the magnitude of hepatic fibrosis, and cause severe functional abnormalities, including thrombocytopenia. Thrombocytopenia heightens the risk of long-term bleeding, limiting the possibility of most treatments and worsening the prognosis (4).

The European Association for the Study of the Liver (EASL) and the American Association for the Study of Liver Disease (AASLD) recommend performing hepatectomy only for patients with HCC without hypersplenism (5, 6). However, patients with good liver function reserve undergo simultaneous hepatectomy and splenectomy, especially in East Asian countries like China and Japan (7, 8). Despite this surgical intervention, the treatment for hypersplenism in HCC patients is still a controversial issue. The spleen is an important functional unit of the immune system, and as the hepatic disease progresses the immune function of the spleen is gradually compensated and resulted in hypersplenism, which further develops dysfunctional and enlarged spleen (9). In such a situation, spleen is always believed to negatively affect the immune function of the liver and the prognosis of HCC (10). However, research evidence on whether hypersplenism affects the prognosis of patients with HCC following hepatectomy is still insufficient due to the heterogeneity of study population and lacking short-term results in some previous studies. Therefore, we carried out this retrospective study of a single center aimed to elucidate the effects of hypersplenism on the prognosis of patients with HBV-related HCC following hepatectomy.

## Methods

### Patients

**Figure 1:**
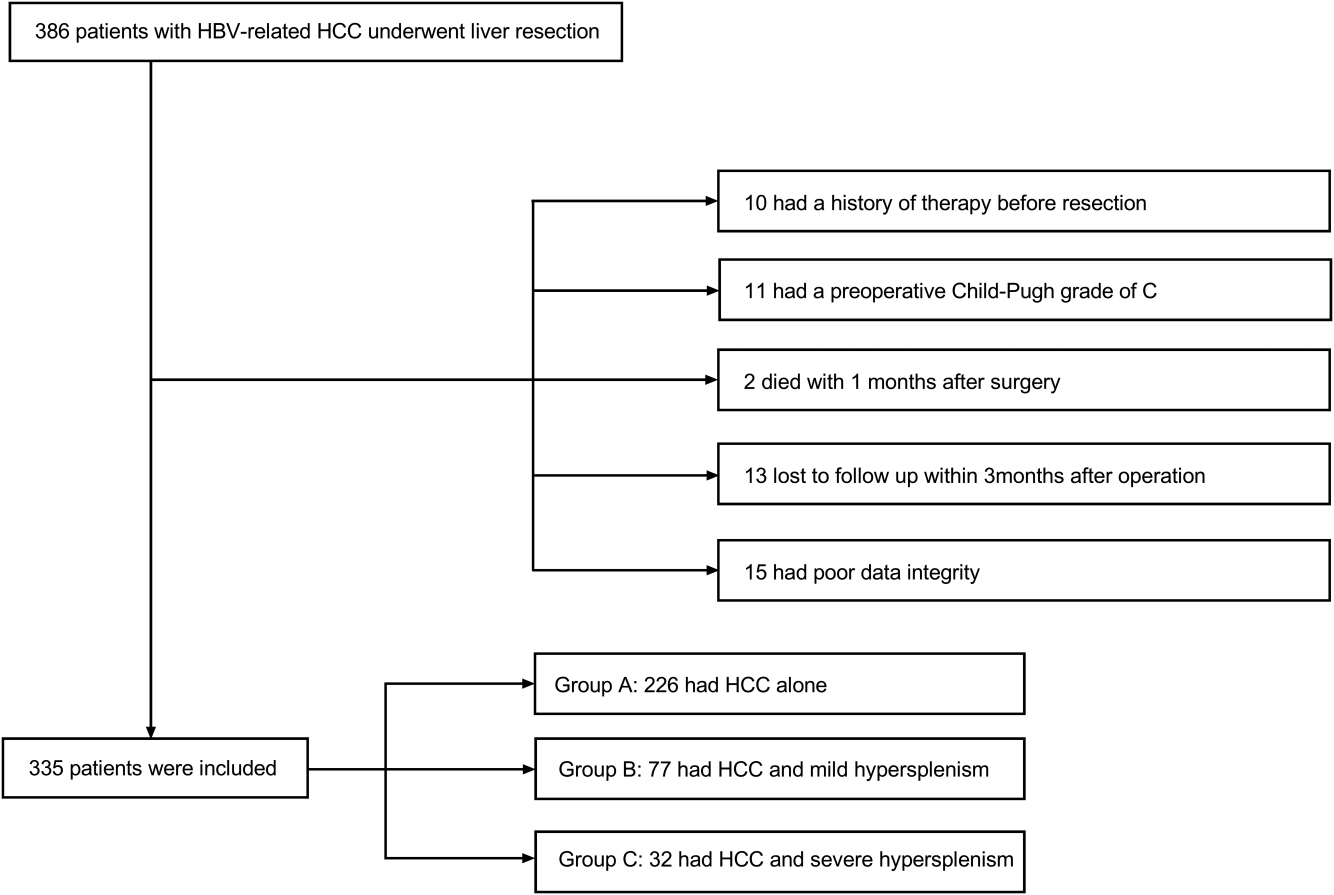
Flowchart of the selection process of study participants for the present analyses.

Data were retrieved for the period from October 2009 to January 2019. A total of 386 patients were diagnosed with hepatocellular carcinoma histopathologically and treated in the general surgery department of the Second Affiliated Hospital of Air Force Military Medical University. All patients underwent hepatectomy without splenectomy for the first line of intervention. Among the patients, 24 subjects received previous therapies, including transhepatic arterial chemotherapy and embolization (TACE) or radiofrequency ablation (RFA). Eleven patients had a Child-Pugh score of grade C, two patients died within one month after surgery, 23 patients failed follow-up examinations within the first 3 months after liver resection, and 15 patients had poor quality data. These patients were all excluded from the study. Eventually, a retrospective study of 335 patients was enrolled. Patients were categorized into 3 groups for further analysis. Group A consisted of 226 patients without hypersplenism. Group B included 77 patients with slight to mild hypersplenism. The remaining 32 patients with severe hypersplenism (a platelet count of less than 50 × 109/L and/or a WBC count of less than 2.0 × 109/L classified the condition as severe hypersplenism) (11) were included in Group C. This proportional distribution was in line with the actual incidence of the clinical situation. Patient selection is summarized in Figure 1. Inclusion criteria were male or female patients with the age between 18 and 75 years, patients with hypersplenism that was defined with a platelet count of less than 100 × 109/L and/or a WBC count of less than 3.0 × 109/L (12), patients with histopathological diagnosis of hepatocellular carcinoma, patients who were positive for hepatitis B surface antigen (HBsAg) and negative for hepatitis C antibody, patients who didn't have any severe heart, lung, brain, or other organ diseases, and patients who didn't have radiological evaluation and diagnosis. Exclusion criteria were patients who didn’t have clinical data, patients with Child-Pugh score of grade C, and patients who were lost to follow-up within one month after discharge from the hospital. This study was approved by the Ethical Committee of Tangdu Hospital-Air Force Medical University (study approval number: 202011–32). This study followed the Declaration of Helsinki principles as well. Written informed consent was waived due to the retrospective nature of the study. The information of all the patients was only used for analytical purposes.

### Data collection

All patient data required for this study were retrieved from the medical records. The basic and clinical variables included in the analysis were age, gender, Child-Pugh score on admission, HBV-DNA levels, preoperative and postoperative antiviral therapies, tumor size and number, and complication with portal hypertension. Perioperative laboratory parameters included in the analysis were blood routine test, coagulation functional evaluation, liver function, serum tumor markers, and Clavien-Dindo Classification of Surgical Complications (13). Perioperative clinical information included in the analysis were operative time, blood loss, intraoperative and postoperative blood transfusions, and postoperative hospital stay.

### Follow-up examination

All patients went through follow-up monitoring one month after the surgery and every 3–6 months thereafter. Follow-up evaluation was conducted by telephone or outpatient visiting for physical examination,, laboratory examinations such as blood routine test and liver function assessment, and imaging examinations were conducted if necessary. When tumor recurrence was clinically suspected, contrast-enhanced computed tomography (CT) and/or MR was performed for definitive diagnosis. Diagnosis of recurrence was based on the initial diagnostic criteria.

The endpoints of the study were overall survival (OS) and disease-free survival (DFS). OS was defined as the time from the date of initial diagnosis until the date of death or last follow-up. DFS was defined as the time from the date of surgery to the recurrence of cancer or death by any cause or the date of the last follow-up. The last follow-up date for this study was July 31st, 2021.

### Statistical analysis

Continuous variables with normal distribution were expressed as mean ± standard deviation, and continuous variables with abnormal distribution were shown as median and interquartile range (IQR). The continuous variables with skewed distribution were compared between the groups using Kruskal-Wallis non-parametric test. Data among the three groups were compared using analysis of covariance (ANCOVA). Categorical variables were classified according to normal reference values or clinical assessment outcomes. Results were compared using the chi-square test or Fisher's exact test.

OS and DFS were analyzed by the Kaplan-Meier method and compared by the log-rank test. Univariate and multivariate analyses of postoperative prognostic factors were carried out using Cox proportional hazards models. Baseline variables deemed clinically relevant or univariately associated with the outcome measures were incorporated into the multivariate Cox proportional hazards regression model. Given the number of events available, the variables included were carefully selected to ensure parsimony in the final model. Two-tailed *P*-values of ≤ 0.05 were considered statistically significant. The IBM SPSS software version 26.0 (IBM Corp., Armonk, NY, United States) and R software (version 4.1.2; R Foundation) was used for all statistical analyses.

## Results

### Baseline characteristics

All patients were histopathologically diagnosed with HCC with hepatitis B viral cirrhosis and received hepatectomy. Furthermore, patients in Group B and Group C manifested in two different degrees of hypersplenism. The basic information for the three groups is shown in Table 1. Most cases from groups A and B had positive for HBV-DNA copies, but only a few cases from group C exhibited positive for the HBV-DNA copies (*P* = 0.001). Group B and Group C had lower albumin (ALB) levels than that in Group A (*P* = 0.005). Due to hypersplenism, spleen-related blood tests manifestations, between Group B and Group C were significantly different [WBC (*P* < 0.001), PLT (*P* < 0.001), PT (*P* < 0.001), and combined portal hypertension (*P* < 0.001)]. The HBV-DNA copies and ALB levels were corrected to the levels before surgery. Furthermore, other baseline characteristics were comparable across groups.

**Table 1:** Baseline characteristics.

Variables	Group A (*n* = 226)	Group B (*n* = 77)	Group C (*n* = 32)	*P*
Age (years)	51.88 ± 10.50	52.36 ± 9.44	50.88 ± 12.40	0.795
Gender				0.400
Male	191 (84.5%)	60 (77.9%)	26 (81.3%)	
Female	35 (15.5%)	17 (22.1%)	6 (18.7%)	
HBV-DNA level (copies/ml)				0.001
Negative	101 (44.7%)	38 (49.4%)	26 (81.3%)	
*P*ositive	125 (55.3%)	39 (50.1%)	6 (18.7%)	
Child-Pugh Classification				0.055
A	215 (95.1%)	68 (88.3%)	28 (87.5%)	
B	11 (4.9%)	9 (11.7%)	4 (12.5%)	
Preoperative antiviral therapy				0.154
No	158 (69.9%)	47 (61.0%)	18 (56.3%)	
Yes	68 (30.1%)	30 (39.0%)	14 (43.7%)	
Postoperative antiviral therapy				0.057
No	82 (36.3%)	35 (45.5%)	18 (56.3%)	
Yes	144 (63.7%)	42 (54.5%)	14 (43.7%)	
AFP (ng/ml)				0.347
≤ 400	150 (66.4%)	51 (66.2%)	17 (53.1%)	
> 400	76 (33.6%)	26 (33.8%)	15 (46.9%)	
ICG-R15 (%) (*n*, %)	6.42 ± 5.34 (200, 88.5)	8.36 ± 5.77 (63, 81.8)	8.84 ± 9.81 (30, 93.8)	0.054
Tumor size (cm)				0.146
≤ 5	105 (46.5%)	44 (57.1%)	19 (59.4%)	
> 5	121 (53.5%)	33 (42.9%)	13 (40.6%)	
Tumor number				0.388
Single	194 (85.8%)	68 (88.3%)	25 (78.1%)	
Multiple	32 (14.2%)	9 (11.7%)	7 (21.9%)	
Portal hypertension				<0.001
No	139 (61.5%)	7 (9.1%)	1 (3.1%)	
Yes	87 (38.5%)	70 (90.9%)	31 (96.9%)	
Extent of liver resection				0.061
1 Liver segment	109 (48.2%)	51 (66.2%)	20 (62.5%)	
2 Liver segments	59 (26.1%)	18 (23.4%)	8 (25.0%)	
3 Liver segments	22 (9.7%)	4 (5.2%)	2 (6.3%)	
Multiple liver segments	36 (15.9%)	4 (5.2%)	2 (6.3%)	
WBC (10^9^/L)	6.09 ± 2.02	4.03 ± 1.56	3.17 ± 1.30	<0.001
PLT (10^9^/L)	174.27 ± 57.33	91.77 ± 35.11	49.94 ± 14.54	<0.001
ALT (IU/L)	41 (26, 55)	39 (24, 49)	41 (28, 43)	0.467
AST (IU/L)	35 (25, 53)	36 (26, 49)	37 (23, 47)	0.873
ALB (g/L)	40.78 ± 4,70	39.76 ± 4.61	38.08 ± 3.90	0.005
TBIL (*μ*mol/L)	15.63 ± 7.88	18.50 ± 9.09	21.94 ± 9.78	0.743
PT (s)	11.74 ± 1.17	12.06 ± 1.16	12.63 ± 1.13	<0.001
AJCC TNM stage (8th)				0.953
I	160 (70.8%)	56 (72.7%)	23 (71.9%)	
II	15 (6.6%)	5 (6.5%)	3 (9.4%)	
III	51 (22.6%)	16 (20.8%)	6 (18.7%)	
CNLC stage				0.992
I	174 (77.0%)	59 (76.6%)	25 (78.1%)	
II	19 (8.4%)	7 (9.1%)	3 (9.4%)	
III	33 (14.6%)	11 (14.3%)	4 (12.5%)	
BCLC stage				0.451
0/A	88 (38.9%)	37 (48.1%)	17 (53.1%)	
B	104 (46.0%)	29 (37.7%)	11 (34.4%)	
C	34 (15.1%)	11 (14.2%)	4 (12.5%)	

HBV, hepatitis B virus; AFP, alpha-fetoprotein; ICG-R15, indocyanine green retention rate at 15 min; WBC, white blood cell; PLT, platelet; ALT, alanine aminotransferase; AST, aspartate aminotransferase; ALB, albumin; TBIL, total bilirubin; PT, prothrombin time; AJCC, american joint committee on cancer; TNM, tumor, node, metastases; CNLC, China liver cancer staging; BCLC, barcelona clinic liver cancer.

Normal continuous variables were compared using ANCOVA. Nonnormal continuous variables were compared using the Kruskal-Wallis nonparametric test. Categorical variables were compared using the chi-square test or Fisher's exact test.

### Perioperative data

A summary of perioperative variables is shown in Table 2. During the surgical procedure, no significant differences in blood loss and operation time among these three groups were observed. But, patients in Group C required more postoperative blood transfusions (*P* = 0.025) and longer hospital stay (*P* = 0.012).

**Table 2:** Major complication classification and other clinical data.

Variables	Group A (*n* = 226)	Group B (*n* = 77)	Group C (*n* = 32)	*P*
Operative time (min)	210 (165, 265)	217 (165, 260)	191 (178, 240)	0.685
Intraoperative bleeding (ml)	400 (300, 800)	400 (200, 700)	400 (275, 625)	0.532
Hospital stays (days)	9 (7, 11)	9 (7, 11)	11 (8, 13)	0.012
Postoperative blood transfusion				0.025
No	191 (84.5%)	60 (77.9%)	21 (65.6%)	
Yes	35 (15.5%)	17 (22.1%)	11 (34.4%)	
With complication of Clavien-Dindo grade IIIA or above				
Total	10 (4.4%)	9 (11.5%)	10 (30.3%)	<0.001
IIIA	6	5	5	
IIIB	1	1	1	
IVA	2	1	2	
IVB	1	1	1	
V	0	1	1	

Grade III A: group A, 4 patients received abdominocentesis and 2 underwent pleurocentesis; group B, 2 patients received abdominocentesis and 3 underwent pleurocentesis; group C, 4 patients received abdominocentesis and 1 underwent pleurocentesis.

Grade IIIB: group A, 1 patient underwent re-laparotomy for intra-abdominal bleeding; group B, 1 patient underwent re-laparotomy for intra-abdominal bleeding; group C, 1 patient underwent re-laparotomy for intra-abdominal bleeding.

Grade IVA: group A, 1 patient needed ICU management due to the renal failure and 1 due to the pulmonary embolism; group B, 1 patient needed ICU management due to the hypotension and shock; group C, 1 patient needed ICU management due to the liver failure.

Grade IVB: group A, 1 patient needed ICU management due to the hepatorenal syndrome; group B, 1 patient needed ICU management due to the hepatorenal syndrome; group C, 1 patient needed ICU management due to the hepatorenal syndrome.

Grade V: group A, no patient died during the perioperative period; group B, 1 patient died of septic shock post-discharge within one months; group C, 1 patient died of intra-abdominal bleeding post-discharge within one months.

Nonnormal continuous variables were compared using the Kruskal-Wallis nonparametric test. Categorical variables were compared using the chi-square test or Fisher's exact test.

For postoperative complications, major complications were defined as complications of grade III or above and included in this study. Ten cases (4.4%) in Group A, nine cases (11.5%) in Group B, and ten cases (30.3%) in Group C developed complications of grade III or above. The differences in the complication category were significant among the three groups (*P* < 0.001).

### Survival rate and prognostic factors

**Figure 2:**
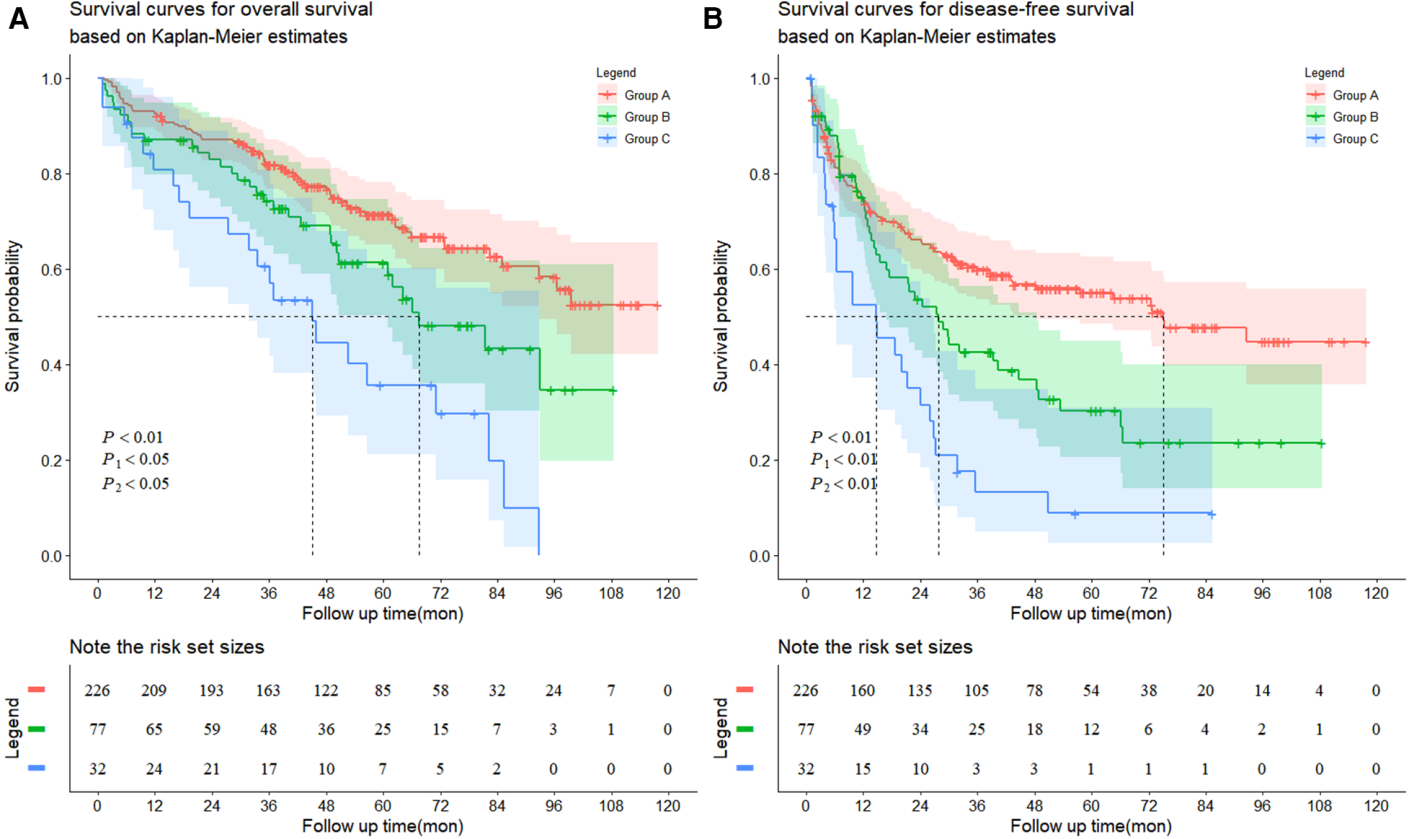
Kaplan-Meier survival curves for (**A**) overall survival and (**B**) disease-free survival. (**A**) The patients exhibited HCC without hypersplenism in the group A had much better overall survival rate than the patients with HCC and mild hypersplenism in the group B (the 1-, 3-, 5-, 7-, and 9-year overall survival rates: 92.5%, 81.8%, 71.3%, 62.5% and 52.5%, respectively vs. 87.0%, 74.2%, 61.3%, 43.2% and 34.6%, respectively, *P*1 = 0.0201). The patients in the group B had much better overall survival rate than the patients with HCC and severe hypersplenism in the group C (the 1-, 3-, 5-, 7-, and 9-year overall survival rates: 87.0%, 74.2%, 61.3%, 43.2% and 34.6%, respectively vs. 80.8%, 60.6%, 35.6%, 19.8% and 0%, respectively, *P*2 = 0.0144). (**B**) The patients exhibited HCC without hypersplenism in the group A had much better disease-free survival rate than the patients with HCC and mild hypersplenism in the group B (the 1-, 3-, 5-, 7-, and 9-year overall survival rates: 74.2%, 59.9%, 55.0%, 47.7% and 44.7%, respectively vs. 75.1%, 42.6%, 30.3%, 23.5% and 23.5%, respectively, *P*1 = 0.0053). The patients in the group B had much better disease-free survival rate than the patients with HCC and severe hypersplenism in the group C (the 1-, 3-, 5-, 7-, and 9-year overall survival rates: 75.1%, 42.6%, 30.3%, 23.5% and 23.5%, respectively vs. 53.4%, 13.1%, 8.7%, 8.7% and 8.7%, respectively, *P*2 = 0.0049).

The follow-up data are shown in Table 3 and Figure 2. The average follow-up time was 64.68 ± 1.65 months. The average OS (*P* < 0.001) and DFS (*P* < 0.001) were significantly different among the three groups. Similarly, significant differences in the number of recurrences (*P* < 0.001) and deaths (*P* < 0.001) were found. In addition, from the follow-up data, HBV-DNA was negative in all patients after six months. Group A had significantly better OS than Group B (*P* = 0.020), and Group B had significantly better OS than Group C (*P* = 0.014) (Figure 2A). Similarly, Group A had the best DFS, followed by Group B (*P* = 0.005, *P* = 0.005) (Figure 2B).

**Table 3:** Follow-up data.

Variables	Group A (*n* = 226)	Group B (*n* = 77)	Group C (*n* = 32)	*P*
**Follow-up duration (months)**	65.09 ± 1.91	62.38 ± 3.54	66.28 ± 5.92	0.614
**Overall survival time (months)**	85.44 ± 3.142	68.09 ± 4.96	47.58 ± 5.98	<0.001
**Disease-free survival time (months)**	67.37 ± 3.71	43.57 ± 5.18	21.35 ± 4.45	<0.001
**No. of recurrence**	** **			<0.001
No	127 (56.2%)	30 (39.0%)	6 (18.8%)	
Yes	99 (43.8%)	47 (61.0%)	26 (81.2%)	
**No. of death**	** **			<0.001
No	157 (69.5%)	44 (57.1%)	10 (31.2%)	
Yes	69 (30.5%)	33 (42.9%)	22 (68.8%)	
**HBV-DNA level (copies/ml)**	** **			1.000
Negative	226 (100.0%)	77 (100.0%)	32 (100.0%)	
Positive	0 (0.0%)	0 (0.0%)	0 (0.0%)	
**Disease-free survival**	** **			<0.001
One-year DFS rate (%)	74.2%	75.1%	53.4%	
Three-year DFS rate (%)	59.9%	42.6%	13.1%	
Five-year DFS rate (%)	55.0%	30.3%	8.7%	
Seven-year DFS rate (%)	47.7%	23.5%	8.7%	
Nine-year DFS rate (%)	44.7%	23.5%	8.7%	
**Overall survival**	** **			<0.001
One-year OS rate (%)	92.5%	87.0%	80.8%	
Three-year OS rate (%)	81.8%	74.2%	60.6%	
Five-year OS rate (%)	71.3%	61.3%	35.6%	
Seven-year OS rate (%)	62.5%	43.2%	19.8%	
Nine-year OS rate (%)	52.5%	34.6%	0.0%	

OS, overall survival; DFS, disease-free survival.

Normal continuous variables were compared using ANCOVA. Categorical variables were compared using the chi-square test or Fisher's exact test. Survival data were compared using the log-rank test.

Table 4 shows prognosis-related risk factors derived from univariate and multivariate Cox regression analyses. Multivariate analysis showed that mild hypersplenism (HR = 2.063, 95% CI: 1.351–3.151, *P* < 0.001), severe hypersplenism (HR = 3.754, 95% CI: 2.241–6.287, *P* < 0.001), positive HBV-DNA copies (HR = 1.571, 95% CI: 1.073–2.300, *P* = 0.020), serum AFP levels of >400 ƞg/ml (HR = 1.950, 95% CI: 1.335–2.848, *P* < 0.001), and tumor size of >5 cm (HR = 2.215, 95% CI: 1.482–3.310, *P* < 0.001) were important independent prognostic factors for OS in patients with HBV-related HCC. Furthermore, in multivariate analysis of DFS, mild hypersplenism (hazard ratio [HR] = 1.778, 95% confidence interval [CI]: 1.250–2.529, *P* = 0.001), severe hypersplenism (HR = 3.183, 95% CI: 2.057–4.926, *P* < 0.001), and tumor size of >5 cm (HR = 1.635, 95% CI: 1.206–2.215, *P* = 0.002) were independent prognostic factors in patients with HBV-related HCC. As the severity of hypersplenism increased, the risk of death and recurrence rose in patients with HBV- related HCC.

**Table 4:** Univariate and multivariate analyses of prognostic factors for disease-free survival and overall survival.

Variables	Disease-free survival				Overall survival			
	Univariate analysis		Multivariate analysis		Univariate analysis		Multivariate analysis	
	HR (95% CI)	*P*	HR (95% CI)	*P*	HR (95% CI)	*P*	HR (95% CI)	*P*
Gender (M vs. F)	1.198 (0.789–1.821)	0.397			0.921 (0.590–1.439)	0.719		
Age (>50 vs. ≤ 50 years)	0.703 (0.521–0.949)	0.021			0.773 (0.543–1.100)	0.153		
Preoperative antiviral therapy (yes vs. no)	0.849 (0.615–1.172)	0.319			0.622 (0.408–0.949)	0.028		
Postoperative antiviral therapy (yes vs. no)	0.917 (0.677–1.242)	0.576			0.556 (0.389–0.796)	0.001		
HBV-DNA (positive vs. negative)	1.136 (0.841–1.533)	0.406			1.316 (0.916–1.891)	0.137	1.571 (1.073–2.300)	0.020
Child-Pugh Classification (B vs. A)	0.888 (0.482–1.636)	0.704			1.417 (0.798–2.519)	0.234		
AFP (>400 vs. ≤ 400 ng/ml)	1.220 (0.892–1.667)	0.213			2.495 (1.752–3.553)	<0.001	1.950 (1.335–2.848)	0.001
Tumor size (>5 vs. ≤ 5 cm)	1.502 (1.113–2.029)	0.008	1.635 (1.206–2.215)	0.002	2.421 (1.656–3.540)	<0.001	2.215 (1.482–3.310)	<0.001
Number of tumors (multiple vs. single)	1.059 (0.682–1.644)	0.798			1.700 (1.096–2.638)	0.018		
Portal hypertension (yes vs. no)	1.760 (1.286–2.408)	<0.001			1.859 (1.279–2.701)	<0.001		
Hypersplenism		<0.001		<0.001		<0.001		<0.001
No				reference		reference		reference
Mild	1.640 (1.158–2.323)	0.005	1.778 (1.250–2.529)	0.001	1.644 (1.085–2.492)	0.019	2.063 (1.351–3.151)	0.001
Severe	3.042 (1.966–4.707)	<0.001	3.183 (2.057–4.926)	<0.001	3.158 (1.948–5.122)	<0.001	3.754 (2.241–6.287)	<0.001

OS, overall survival; DFS, disease-free survival; HR, hazard ratio; CI, confidence interval.

Univariate and multivariate analyses of prognostic factors were carried out using Cox proportional hazards models.

**Figure 3:**
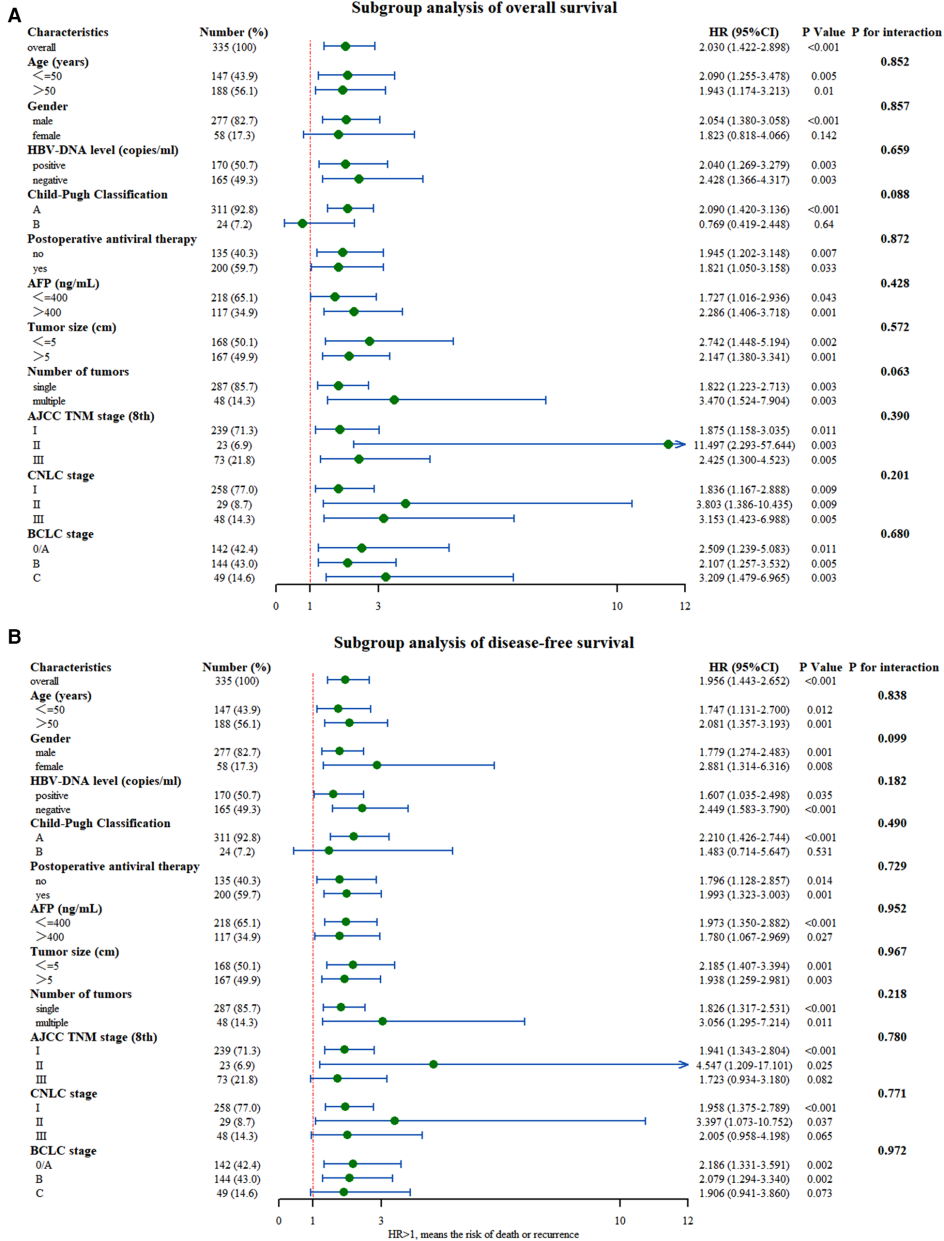
Subgroup analysis of (**A**) overall survival and (**B**) disease-free survival between the two groups with and without hypersplenism.

To further understand the internal consistency, several subgroup analyses of OS and DFS were performed, which were stratified by basic characteristics, tumor factors, and liver function reserve parameters (Figures 3A, B, respectively). Since the *p*-value for interaction was nonsignificant, the effect of hypersplenism on survival wasconsistently negative across all subgroups. Furthermore, the HR values observed in a small subset of subgroups were not significant. Especially in the advanced tumor stage (BCLC C; TNM III; CNCL III), hypersplenism was still the prognostic factor for OS in patients with HBV-related HCC (*P* = 0.003, *P* = 0.005, *P* = 0.005), but not for DFS. Nevertheless, this result should be verified using a study with larger sample size.

## Discussion

HBV infection is an important risk factor for HCC, especially when occurred simultaneously with chronic liver disease and even cirrhosis (14), and these patients often suffered from hypersplenism (15). The treatment of hypersplenism in patients with HCC is controversial. Since severe liver dysfunction and low platelet counts are associated in these cases, managing perioperative bleeding and postoperative liver failure is of the main concern. Sometimes, these cases cannot be surgically managed (16). In addition, the outcome from simultaneous hepatectomy and splenectomy was inconsistent in different studies (12, 17). No unified opinion on the treatment of HBV-related HCC with hypersplenism exists currently. Therefore, this study further aimed to reveal the clinical outcome differences in these patients. We found that hypersplenism significantly worsened the prognosis and need to be treated more actively.

The spleen is an important part of the immune system, but its normal function is affected in the presence of liver cirrhosis and compensatory hypersplenism (18). Jasnis et al. found that the immune function of the spleen was decreased with the progression of liver cancer (19). The spleen can also affect the liver microenvironment through complex liver-spleen cross-talks. Spleen-derived immune cells can directly change the composition of hepatic leukocytes by releasing cytokines or being recruited by the liver, forming an immune microenvironment that is conducive to tumor growth and development (20). On the other hand, tumor-derived factors alter the hepatic microenvironment, gradually increasing the immunosuppressive nature and tumor-stimulating functions of the spleen (20). Some previous studies revealed that the marginal area of the splenic tissue from patients with tumors contained a large number of CD11b + Gr-1intLy6Chi cells, resulting in the abnormality of the T cell receptor CD3 complex, inhibiting T cell immune function, and leading to tumor immune tolerance (21). Our results supported the above findings by exhibiting the severity level of hypersplenism directly associated with prognosis results. Splenectomy in mice eliminated the tumor-induced tolerance by reducing the proportion of myeloid-derived suppressor cells in peripheral blood, restoring lymphocyte function, and increasing the natural killer cells (22). The spleen also affected the progress of liver regeneration through transforming growth factor *β*1 (TGF-*β*1), hepatocyte growth factor (HGF), thrombospondin-1 (TSP-1), and cytokines such as TNF-α and IL-6 (23). Our previous research found that splenectomy attenuated liver fibrosis by reducing the expression of LIGHT [Tumor necrosis factor superfamily 14 (TNFSF14; also known as LIGHT)] (24). It was speculated that hypersplenism showed adverse effects on the prognosis of patients with HCC complicated with cirrhosis. To further explore the effect of hypersplenism on prognosis and reduce bias, we retrieved basic and clinical data from patients who were followed for approximately ten years for retrospective analysis and evaluated potential confounding variables that might have affected the prognosis.

The baseline data was inconsistent with HBV-DNA copies and ALB levels as determined from our study. The patient with hypersplenism might possibly have decompensated the liver cirrhosis very early and received antiviral therapy earlier. Liver cancer occurring under this condition usually indicating a poor hepatic function. Patients with HCC without hypersplenism often compensated for liver cirrhosis, but many of them were asymptomatic. Most of these patients did not receive standard antiviral therapy, abut their hepatic function preserve was still acceptable. Thus, patients with hypersplenism had lower albumin levels and their HBV-DNA copies was mostly negative. In most patients who received standard antiviral treatment before surgery, the number of HBV-DNA copies was reduced to a normal level on the 6th-month follow-up. Hypersplenism prolonged hospital stay and increased the frequency of postoperative blood transfusions. Regarding the postoperative complications, complication categories were found statistically differed among the three groups. Since the hypersplenism was not resolved in these cases, the low WBC count, reduced PLT count, and coagulation abnormalities were not improved. The decline in platelet counts can increase the risk of internal and external bleeding (25). Unameliarated hypersplenism might increase the incidence of ascites and deteriorated hepatic function in patients with HCC (26). Therefore, patients with hypersplenism should receive more meticulous perioperative care to promote their recovery. Regarding the long-term outcome, the mean survival time and mean recurrence time were greatly reduced in HCC patients accompanied with hypersplenism, and further decreased with the severity of hypersplenism. On Kaplan-Meier analysis, the OS and DFS in patients with HCC were decreased significantly with the increasing severity of hypersplenism, and they were significantly different among the groups. This result may have been due to the postoperative inflammatory response caused by hypersplenism, and systemic inflammation that was associated with poor prognosis in HCC. Tumor cells proliferate and metastasize by promoting angiogenesis, damaging DNA structure and function, inhibiting apoptosis, and evading immunosurveillance *via* inflammation. Therefore, aggressive treatment of postoperative inflammation may be critical for improving the prognosis of HCC patients. Furthermore, liver function damage and even liver failure may occur in patients with HCC and hypersplenism after surgery (27), leading to poor OS.

Multivariate analysis revealed that hypersplenism and tumor size of >5 cm were found to be independent prognostic factors for both OS and DFS among patients with HBV-related HCC. Nevertheless, positive HBV-DNA copies and serum AFP levels of >400 ƞg/ml were independent prognostic factors only for OS. Factors other than hypersplenism were similar in the groups at baseline. Specific to tumor size, the patients with tumor size greater than 5 cm had a significantly worse prognosis (28) because vascular infiltration and tumor differentiation were closely related to the size of HCC (29). In addition to the effects of hypersplenism on tumor immune tolerance, hemodynamic abnormalities resulting from hypersplenism can induce abnormal liver function, influencing the outcome (4). Platelet counts were decreased significantly in hypersplenism, and thrombocytopenia aggravated the progression of liver abnormalities (30). Platelets stimulated the proliferation of liver sinusoidal endothelial cells (LSEC) and accelerated the DNA synthesis in hepatocytes (31). Furthermore, platelets can directly interact with hepatocytes to induce hepatic regeneration. Coculture experiments found that platelets that are in contact with hepatocytes triggered the secretion of growth factors, including IGF-1, HGF, and VEGF, and thus induced the proliferation of hepatocytes (32). But in contrast to mice, human platelets fail to secrete enough HGF. Therefore, this mechanism may not apply to humans. As for the role of the spleen in patient prognosis, a retrospective study with long-term follow-up by Zhang et al. showed consistent results with our study (12). But the definition of the pathological spleen as splenomegaly and hypersplenism in this article was different from that in our study. Our study removed this bias and targeted the investigation of hypersplenism. As the severity of hypersplenism increased, laboratory parameters were further affected. The negative regulation of the liver was enhanced, the beneficial effects diminished, and the imbalance between them became more severe. This outcome was consistent with our results, and both mild and severe hypersplenism were found to be independent risk factors for OS and DFS.

Subgroup analysis showed good internal consistency for the effect of hypersplenism on survival. Although statistically significant, the *p*-values associated with HR at different subgroup levels should be interpreted with caution (33). For example, they were female patients and with Child-Pugh B in the OS analysis, and patients with advanced tumor stage (BCLC C; TNM III; CNCL III) and patients with Child-Pugh B in DFS analysis. The associated *P*-values for HR in the above groups were not significant. This insignificance could be explained by the intergroup variability, which may have increased the proportion of patients with extreme values or with short follow-up time. This may have resulted in the inability to draw an accurate conclusion. Patients with advanced tumor stage and poor liver function had poor prognoses regardless of the cooccurrence of HCC and hypersplenism (34). Thus, the effect of hypersplenism on prognosis in these patients might have been masked, making the conclusion insignificant. Hence, the intervention on the spleen should be carried out as early as possible. It is uncertain whether hypersplenism can reduce the recurrence rate. But some previous studies showed that splenectomy before hepatectomy or simultaneous hepatectomy and splenectomy could both increase the DFS rate of patients with HCC and hypersplenism, while splenectomy before hepatectomy might reduce the hypersplenism without increasing the risk of surgery (35). However, these findings still need further verifications.

This study still has some limitations. First, selection bias may not have been avoided in this type of nonrandomized study. Second, all samples came from a single medical center. Third, few cases didn’t receive complete follow-up due to the study time limitation. Therefore, expansion of the sample size or randomized controlled trial should be carried out to investigate the influence of hypersplenism on patients with HCC and hepatitis B cirrhosis following hepatectomy.

In conclusion, hypersplenism affected perioperative and postoperative results and prognosis in patients with HBV-related HCC. The hypersplenism prolonged the hospital stays, increased the rate of postoperative blood transfusions, and increased the incidence of complications. With the increasing severity of hypersplenism, the prognosis of patients gradually deteriorated. Additionally, severity of hypersplenism could be used as an important prognostic factor for tumor recurrence and long-term survival of patients.

## Data Availability

The raw data supporting the conclusions of this article will be made available by the authors, without undue reservation.

## References

[1] Bray F Ferlay J Soerjomataram I Siegel RL Torre LA Jemal A. Global cancer statistics 2018: gLOBOCAN estimates of incidence and mortality worldwide for 36 cancers in 185 countries. CA Cancer J Clin. (2018) 68(6):394–424. 10.3322/caac.21492 30207593

[2] Petrick JL Florio AA Znaor A Ruggieri D Laversanne M Alvarez CS International trends in hepatocellular carcinoma incidence, 1978–2012. Int J Cancer. (2020) 147(2):317–30. 10.1002/ijc.32723 31597196 PMC7470451

[3] Sherman M Bruix J Porayko M Tran T Committee APG. Screening for hepatocellular carcinoma: the rationale for the American association for the study of liver diseases recommendations. Hepatology. (2012) 56(3):793–6. 10.1002/hep.25869 22689409

[4] Bosch J Iwakiri Y. The portal hypertension syndrome: etiology, classification, relevance, and animal models. Hepatol Int. (2018) 12(Suppl 1):1–10. 10.1007/s12072-017-9827-9 29064029

[5] Marrero JA Kulik LM Sirlin CB Zhu AX Finn RS Abecassis MM Diagnosis, staging, and management of hepatocellular carcinoma: 2018 practice guidance by the American association for the study of liver diseases. Hepatology. (2018) 68(2):723–50. 10.1002/hep.29913 29624699

[6] European Association for the study of the L, European organisation for R, treatment of C. EASL-EORTC clinical practice guidelines: management of hepatocellular carcinoma. J Hepatol. (2012) 56(4):908–43. 10.1016/j.jhep.2011.12.001 22424438

[7] Belli A Cioffi L Russo G Belli G. Liver resection for hepatocellular carcinoma in patients with portal hypertension: the role of laparoscopy. Hepatobiliary Surg Nutr. (2015) 4(6):417–21. 10.3978/j.issn.2304-3881.2015.12.02 26734627 PMC4689686

[8] Ishizawa T Hasegawa K Aoki T Takahashi M Inoue Y Sano K Neither multiple tumors nor portal hypertension are surgical contraindications for hepatocellular carcinoma. Gastroenterology. (2008) 134(7):1908–16. 10.1053/j.gastro.2008.02.091 18549877

[9] Wei W Li L Kong G Zheng Z Ji F Li Z. Spleen in hepatocellular carcinoma: more complexity and importance than we knew. J Hepatol. (2019) 70(4):805–6. 10.1016/j.jhep.2018.11.022 30683601

[10] Nomura Y Kage M Ogata T Kondou R Kinoshita H Ohshima K Influence of splenectomy in patients with liver cirrhosis and hypersplenism. Hepatol Res. (2014) 44(10):E100–9. 10.1111/hepr.12234 24033833

[11] Zhu J Zhu H Mei Z Jin C Ran L Zhou K High-intensity focused ultrasound ablation for treatment of hepatocellular carcinoma and hypersplenism: preliminary study. J Ultrasound Med. (2013) 32(10):1855–62. 10.7863/ultra.32.10.1855 24065267

[12] Zhang X Li C Wen T Peng W Yan L Li B Synchronous splenectomy and hepatectomy for patients with small hepatocellular carcinoma and pathological spleen: neutrophil to lymphocyte ratio changes can predict the prognosis. Oncotarget. (2017) 8(28):46298–311. 10.18632/oncotarget.17758 28549349 PMC5542268

[13] Dindo D Demartines N Clavien PA. Classification of surgical complications: a new proposal with evaluation in a cohort of 6336 patients and results of a survey. Ann Surg. (2004) 240(2):205–13. 10.1097/01.sla.0000133083.54934.ae 15273542 PMC1360123

[14] Lam CM Chan AO Ho P Ng IO Lo CM Liu CL Different presentation of hepatitis B-related hepatocellular carcinoma in a cohort of 1863 young and old patients—implications for screening. Aliment Pharmacol Ther. (2004) 19(7):771–7. 10.1111/j.1365-2036.2004.01912.x 15043518

[15] Chen W Zheng R Baade PD Zhang S Zeng H Bray F Cancer statistics in China, 2015. CA Cancer J Clin. (2016) 66(2):115–32. 10.3322/caac.21338 26808342

[16] Bruix J Sherman M. American Association for the study of liver D. Management of hepatocellular carcinoma: an update. Hepatology. (2011) 53(3):1020–2. 10.1002/hep.24199 21374666 PMC3084991

[17] Shi R Zhang YM Zhu ZJ Deng YL Pan C Zheng H Synchronous splenectomy and hepatectomy in patients with hepatocellular carcinoma, hypersplenism and liver cirrhosis. Hepatogastroenterology. (2014) 61(133):1363–7. 25436312

[18] Asanoma M Ikemoto T Mori H Utsunomiya T Imura S Morine Y Cytokine expression in spleen affects progression of liver cirrhosis through liver-spleen cross-talk. Hepatol Res. (2014) 44(12):1217–23. 10.1111/hepr.12267 24506078

[19] Jasnis MA Eljan AM Oisgold-Daga S. Regulation of tumor growth by soluble spleen factors: effect of tumor resection. J Surg Oncol. (1987) 35(2):139–45. 10.1002/jso.2930350216 3586684

[20] Han Y Liu Q Hou J Gu Y Zhang Y Chen Z Tumor-Induced generation of splenic erythroblast-like ter-cells promotes tumor progression. Cell. (2018) 173(3):634–48.e12. 10.1016/j.cell.2018.02.061 29606356

[21] Ugel S Peranzoni E Desantis G Chioda M Walter S Weinschenk T Immune tolerance to tumor antigens occurs in a specialized environment of the spleen. Cell Rep. (2012) 2(3):628–39. 10.1016/j.celrep.2012.08.006 22959433

[22] Huang N Ji FP Zhang S Li Z Li J Zhou R Spleen-Associated effects on immunity in hepatitis B virus-related cirrhosis with portal hypertension. J Interferon Cytokine Res. (2019) 39(2):95–105. 10.1089/jir.2018.0121 30676849

[23] Liu G Xie C Fang Y Qian K Liu Q Liu G Splenectomy after partial hepatectomy accelerates liver regeneration in mice by promoting tight junction formation via polarity protein par 3-aPKC. Life Sci. (2018) 192:91–8. 10.1016/j.lfs.2017.11.032 29166570

[24] Liang QS Xie JG Yu C Feng Z Ma J Zhang Y Splenectomy improves liver fibrosis via tumor necrosis factor superfamily 14 (LIGHT) through the JNK/TGF-beta1 signaling pathway. Exp Mol Med. (2021) 53(3):393–406. 10.1038/s12276-021-00574-2 33654222 PMC8080781

[25] Ng SSM Nagy BA Jensen SM Hu X Alicea C Fox BA Heterodimeric IL15 treatment enhances tumor infiltration, persistence, and effector functions of adoptively transferred tumor-specific T cells in the absence of lymphodepletion. Clin Cancer Res. (2017) 23(11):2817–30. 10.1158/1078-0432.CCR-16-1808 27986749 PMC5805388

[26] Dib N Oberti F Cales P. Current management of the complications of portal hypertension: variceal bleeding and ascites. CMAJ. (2006) 174(10):1433–43. 10.1503/cmaj.051700 16682712 PMC1455434

[27] Dimick JB Cowan JA Jr. Knol JA Upchurch GR Jr. Hepatic resection in the United States: indications, outcomes, and hospital procedural volumes from a nationally representative database. Arch Surg. (2003) 138(2):185–91. 10.1001/archsurg.138.2.185 12578418

[28] Pelizzaro F Penzo B Peserico G Imondi A Sartori A Vitale A Monofocal hepatocellular carcinoma: how much does size matter? Liver Int. (2021) 41(2):396–407. 10.1111/liv.14718 33155401

[29] Kim SJ Lee KK Kim DG. Tumor size predicts the biological behavior and influence of operative modalities in hepatocellular carcinoma. Hepatogastroenterology. (2010) 57(97):121–6. 20422886

[30] Kodama T Takehara T Hikita H Shimizu S Li W Miyagi T Thrombocytopenia exacerbates cholestasis-induced liver fibrosis in mice. Gastroenterology. (2010) 138(7):2487–98. 10.1053/j.gastro.2010.02.054 20206174

[31] Kawasaki T Murata S Takahashi K Nozaki R Ohshiro Y Ikeda N Activation of human liver sinusoidal endothelial cell by human platelets induces hepatocyte proliferation. J Hepatol. (2010) 53(4):648–54. 10.1016/j.jhep.2010.04.021 20615569

[32] Matsuo R Ohkohchi N Murata S Ikeda O Nakano Y Watanabe M Platelets strongly induce hepatocyte proliferation with IGF-1 and HGF in vitro. J Surg Res. (2008) 145(2):279–86. 10.1016/j.jss.2007.02.035 17688880

[33] Barraclough H Govindan R. Biostatistics primer: what a clinician ought to know: subgroup analyses. J Thorac Oncol. (2010) 5(5):741–6. 10.1097/JTO.0b013e3181d9009e 20421767

[34] Llovet JM Burroughs A Bruix J. Hepatocellular carcinoma. Lancet. (2003) 362(9399):1907–17. 10.1016/S0140-6736(03)14964-1 14667750

[35] Zhou C Huang Y Shu C Zhou J Hu X Wang J Splenectomy before hepatectomy for patients with hepatocellular carcinoma and hypersplenism: a retrospective study. Medicine (Baltimore). (2021) 100(4):e24326. 10.1097/MD.0000000000024326 33530224 PMC7850697

